# Health care experiences of U.S. Retirees living in Mexico and Panama: a qualitative study

**DOI:** 10.1186/1472-6963-13-411

**Published:** 2013-10-12

**Authors:** Philip D Sloane, Lauren W Cohen, Bryce E Haac, Sheryl Zimmerman

**Affiliations:** 1 Program on Aging, Disability, and Long-Term Care, Cecil G. Sheps Center for Health Services Research, University of North Carolina, Chapel Hill, NC 27514, USA; 2 Department of Family Medicine, University of North Carolina, Chapel Hill, NC 27514, USA; 3 The School of Social Work, University of North Carolina, Chapel Hill, NC 27514, USA

**Keywords:** Retirement, Migration, Medical care, Mexico, Panama

## Abstract

**Background:**

Retirement migration from northern countries to southern countries is increasing in both Europe and North America, and retiree experiences will impact future migration and health services utilization. We therefore sought to describe the healthcare experiences and perceptions of retired U.S. citizens currently living in Mexico and Panama.

**Methods:**

46 retired U.S. citizens (23 per country) who had been hospitalized (61%) or had a chronic health condition (78%) in two regions per country with large communities of retired U.S. citizens were identified. Detailed semi-structured interviews were conducted to explore experiences with, attitudes toward, and costs of healthcare. Interviews were analyzed using quantitative and qualitative methods.

**Results:**

Respondents averaged 68–70 years old, were well educated, had few physical dependencies, and had moderate incomes. They praised physician services as more personalized than in the U.S. and home care as inexpensive and widely available, expressed favorable opinions regarding outpatient and dental care, gave mixed ratings on hospital services, and expressed concerns about emergency services. Numerous concerns about health insurance were expressed, including the unavailability of Medicare and reductions in Tricare. Payment concerns and lack of data on local health providers made deciding where to obtain services challenging.

**Conclusions:**

Retirees living abroad report dilemmas regarding healthcare choices, insurance availability, and quality of care. As this population segment grows, pressure will increase for policy and business solutions to existing medical care challenges.

## Background

Trans-national migration of retirees is an increasingly common phenomenon in Europe and the United States. The common European pattern involves retirees from northern countries such as Germany, the Netherlands, Great Britain, Norway, and Sweden relocating to southern countries such as France, Italy, Spain, and the Canary Islands [1]. In the Western hemisphere, a similar pattern has evolved, with Canadian and U.S. citizens increasingly selecting retirement destinations in Latin America [2,3]. Reasons most commonly offered for migration include warmer climate, lower living costs, and a desire to experience another culture [1,2,4,5].

As of 2011, an estimated 6.32 million non-military American citizens lived overseas, about half of whom live in the Western Hemisphere [6]. Of these, a significant and increasing proportion are retirees living in Latin America, with Mexico and Panama currently being the most popular destinations [2,3]. In 2012, the U.S. Social Security Administration reported that nearly 360,000 retired workers were receiving benefits abroad, a 20 percent increase since 2007 [7]. With the upcoming retirement of over 100 million U.S. “baby boomers” in the next 30 years, many of whom have limited retirement savings and an increasing number of whom are considering non-traditional retirement alternatives, the number of U.S. citizens retiring in Latin America has been projected to mushroom [5,8].

Health care is an important issue for older persons; however health care issues among retirees who migrate abroad have received relatively little attention in the retirement migration literature [1]. For European migrants, medical care is usually reimbursed by national health insurance plans that pay for emergency and planned treatment anywhere in the European Union [9]; however satisfaction with services has been reported to be mixed [10], and major health events can be unusually disruptive [11]. For U.S. migrants, the issue of healthcare abroad is even more challenging, as neither Medicare (the government health insurance for senior citizens) nor most private health insurance plans cover expenses incurred in other countries [12-14]. Therefore, how retired U.S. citizens living abroad manage their healthcare is a particularly important issue [2,8,15,16].

Given the anticipated growth in retirement migration to Mexico and Central America, information about the actual experiences of retirees who have required healthcare while living abroad would help inform personal, corporate, and policy decisions regarding international retirement. Therefore, to learn more about the healthcare experiences and needs of U.S. retirees living abroad, we conducted and analyzed semi-structured interviews of retirees in Mexico and Panama who had used local healthcare resources for acute hospital care, chronic illness management, or both.

## Methods

### Participants and recruitment

In each country, two regions with large numbers of expatriate American retirees were targeted for recruitment, using a purposive sampling approach. In Mexico, recruitment was carried out in San Miguel de Allende and Chapala, both of which are medium-size communities within an hour and a half of large cities. In Panama, recruitment focused on Panama City, the capital, and Boquete, a rural region in the north. Participants were recruited using notices in English and bilingual newspapers, postings to local expatriate blogs and list serves, networking with expatriate societies, and acquaintances of respondents.

Our goal was to interview retirees who required significant use of the local healthcare system. Therefore, participants had to be: native born U.S. citizens who were retired, aged 55 or older, had lived full-time in Mexico or Panama for at least a year, and either have a history of hospitalization in Mexico or Panama during the previous two years or have a chronic illness requiring ongoing medical management and monitoring.

Of 49 eligible volunteers, three were not interviewed, two because the interviewee did not keep the appointment and one who withdrew due to hearing difficulty. The final sample included 23 respondents from Mexico and 23 from Panama. Written informed consent was obtained from all participants, and study procedures were approved by the Institutional Review Board of the University of North Carolina. Ethical approval was not obtained from Mexican and Panamanian authorities since only U.S. Citizens participated in this research. No Mexican or Panamanian nationals were involved in any way in the subject accrual or data collection.

### Data collection

Interviews in Mexico were conducted in March 2007 and in Panama in June and July of 2008; all except for one were conducted in person, and all were audiotaped and transcribed for analysis. Each interview lasted approximately 45 minutes and followed a semi-structured format. Topics addressed included:

• personal health and resources (e.g., functional status; activities of daily living; health status; health insurance);

• healthcare experiences while living abroad (e.g., chronic illness care, serious acute problems, hospitalizations);

• payment for medical care (e.g., out-of-pocket expenses; cost of specific incidents such as hospitalizations or trips back to the U.S. for health care);

• opinions about the quality, availability, and cost of healthcare in the host country (e.g., desires in a health care provider; experiences with providers in the U.S. and Latin America; whether or not they had ever postponed care and if so why);

• future health care plans (e.g., what they would do if they had a serious illness or needed long-term care services); and

• comparison and contrast with the United States (e.g., experiences with health professionals; cost).

Interviews were conducted by trained research assistants with experience in qualitative data collection and health services research, who were encouraged to pursue themes that arose and seek clarification as required.

### Data analysis

Data analysis was conducted using a grounded theory approach [17]. The first step involved developing codes based on the content of each phrase, sentence or paragraph. This was done by four coders from different disciplines (medicine, social work, and psychology) who compared and agreed on a final list of codes. Next, the team members independently coded the transcribed interviews for manifest and latent themes, as outlined by Padgett [18].

Quantitative data were imported into SAS 9.1® and checked for accuracy and logical errors, with discrepancies resolved by reference to the transcribed interview. Frequencies and averages were computed, and analyses compared frequencies and distributions of responses by country. Continuous data were analyzed for significance using the t-test option in SAS 9.1®; categorical data were analyzed using the Chi-Square test or, as appropriate, the Fisher’s Exact option.

## Results

Study respondent characteristics are displayed in Table 1. The mean age was 69.9 for the Mexican respondents and 68.1 for the Panamanian respondents, with the range from 59 to 87. The majority were married, had a college degree, had few dependencies in daily activities, and had some Spanish proficiency. Reported income levels were moderate, with approximately half reporting monthly incomes below $3,000. Twenty-eight respondents (61%) had been hospitalized in Mexico or Panama, and 36 (78%) reported a chronic illness that required ongoing care and monitoring. In general, the two samples did not differ significantly in demographic or health status characteristics, except for greater reported income and fewer white respondents in the Panamanian sample.

**Table 1:** Characteristics of study participants

**Demographic characteristics**	**Mexico (n = 23)**		**Panama (n = 23)**		**P-value for difference**
	**Mean (SD)**	**Range**	**Mean (SD)**	**Range**	
Age	69.9 (6.8)	59.0 – 87.4	68.1 (4.5)	60.8 – 80.6	0.40
Years living in Mexico or Panama	7.2 (5.6)	1.0 – 20.0	6.4 (12.2)	0.6 – 60.6	0.78
Spanish proficiency (0 = none to 10 = native)	4.7 (2.3)	0.0 – 9.0	4.2 (2.4)	0.5 – 8.0	0.51
	**N**	**%**	**N**	**%**	
Gender (female)	15	65.2	8	34.8	0.08
Race (White/Caucasian)	23	100	18	78.3	0.05
Marital status					0.29
Married	14	60.9	17	73.9	
Widowed					
Separated/divorced	6	26.1	5	21.7	
Never married	3	13.0	1	4.4	
Education					0.13
High school or less	3	13.0	0	0.0	
Some college	2	8.7	8	34.8	
College degree	4	17.4	4	17.4	
Post-graduate degree	14	60.9	11	47.8	
Income (monthly)					0.04
$1,000-$1,999	6	26.1	2	8.7	
$2,000-$2,999	8	34.8	8	34.8	
≥ $3,000	9	39.1	13	56.5	
**Medical and functional characteristics**	**N**	**%**	**N**	**%**	
Hospitalized in Mexico or Panama during prior 2 years	11	55.0	17	73.9	0.22
Days hospitalized among participants hospitalized [Mean (SD), Range]*	4.9 (4.8)	1 – 17	3.3 (1.6)	1 – 6	0.36
Had a chronic illness requiring ongoing care	17	77.3	19	82.6	0.72
Requires supervision or assistance with:					
Feeding self	1	4.3	0	0.0	0.48
Walking a block	2	8.7	3	13.0	0.67
Dressing self	2	8.7	0	0.0	0.09
Bathing self	2	8.7	0	0.0	0.09
Using telephone	1	4.3	0	0.0	0.22
Shopping for groceries	2	8.7	0	0.0	0.14
Reliably taking medications	1	4.3	0	0.0	0.19
Handling finances	1	4.3	0	0.0	0.43
Depression	3	13.0	2	8.7	0.61
Incontinence	3	13.0	2	8.7	0.61
Number of medications *[Mean (SD), Range]*	4.9 (2.8)	2 – 12	4 (3.3)	0 – 10	0.39

*Outlier of 75 days removed from Mexican dataset; with outlier, mean (SD) = 11.9 (22.6), p < 0.01.

In qualitative analyses 38 open codes were developed, reflecting four overarching themes: availability, quality and cost of care; relationships with providers; paying for healthcare; and making healthcare choices.

### Availability, quality and cost of care

Both Mexico and Panama have parallel public and private healthcare systems, with the overwhelming majority of interviewees using the private system exclusively or most of the time. Opinions about quality were mixed but generally favorable regarding access, visit length, price, and communication. Negative reports focused on uneven expertise, language difficulties, acute care costs, and less available technology.

#### ***Outpatient care***

Most respondents used the private system for outpatient care, reporting easy access to physicians and generally praised the services received.

A pervasive theme involved doctors providing longer appointments and more personal service than their U.S. counterparts. As one respondent commented: *“You can get an appointment with a specialist, and for 500 pesos they will give you an hour or more of their time, give you their cell phone number, and return your emails.”* Mental healthcare was reported to be less developed than in the U.S., however, and record-keeping was viewed as uneven and often inferior.

#### ***Hospital services***

Nearly all respondents reporting hospitalization had used private hospitals and reported the quality to be good and the facilities adequate. Hospital physicians largely spoke English, and many (especially in Panama) had some U.S. training. Many respondents remarked that the hospital staff provided more time and personalized attention than they had experienced in the U.S. One reported that “*doctors are not frantically busy like they are in the United States*,” and several commented about nursing care being more attentive. A few respondents in Mexico reported choosing to use the public hospital system (the Instituto Mexicano del Seguro Social [IMSS]), which can be purchased by immigrants after a waiting period. IMSS hospitals were reported to have less access to technology than U.S. hospitals, however, and patients were expected to be accompanied by someone who would attend to personal care needs.

For specialized procedures, residents in smaller communities reported having to travel to larger centers, occasionally at considerable cost or hardship. One respondent, for example, had to move from Boquete to Panama City for two months to receive daily radiation treatments.

Persons with Medicare often expressed a desire to go back to the U.S. for hospital care, some citing quality and all citing cost. The risk of delaying care and the cost of travel were cited as significant drawbacks to this strategy, however; one reported spending $13,000 to return to the U.S. by private Lear jet during a health emergency.

A unique aspect of Panamanian healthcare is the availability of a military hospital in Panama City. Unfortunately, veterans in our sample reported problems communicating up the chain of command, because they often had to contact the U.S. to determine whether a given service was covered by their benefits. As one military retiree noted, *“There’s no local direct contact with the VA or Department of Defense or anybody who can give us information….They give us a phone number we can call…(and) there is a whole lot of time involved in trying to communicate by telephone. We get a lot of menus. Sometimes we don’t get an answer at all.”*

#### ***Emergency services***

Respondents in both Mexico and Panama described emergency services as needing improvement, particularly in regard to transport and paramedic care. As one respondent noted, *“what they call paramedics down here are not paramedics; they strictly transport you to the hospital. They cannot administer drugs….Their first aid training is very basic, and they have no requirement to get annual updates.”* The availability of blood supplies in the event of an emergency was also cited as a cause for concern. However, efforts were reportedly being made to improve emergency services in both countries, often with assistance from retirees or U.S. based physicians.

#### ***Long-term care***

In both countries home care was reported to be widely available, inexpensive, and of high quality. *“Where I live, I can get a nurse to take care of me 24 hours a day for about $20 to $25 a day,”* one respondent said. Several employed full-time home care aides, often referring to them as friends and knowing their entire family. As a result, a common theme among many respondents was of planning to remain at home with services until they died.

Few respondents knew about the availability of institutional long-term care. Some had not given it much thought. Others, especially those with close ties to children in the states, reported that they would return to the U.S. if they needed long-term care.

#### ***Dental care***

Dental services in both countries were widely praised for high quality and low cost, except in rural Boquete where it was less available. Comments included “*I know a lot of people who come to Mexico regularly to get their dental care,*” and “*you wouldn’t get a cleaning in the U.S. anywhere near the quality of this*.”

#### ***Medication***

Respondents in both countries reported that most medications other than narcotics were available without prescription. They tended to appreciate the easy access, but some reported concern about potential use of unnecessary or contraindicated medications. Costs varied and were reported to be increasing. As a result, interviewees with U.S. insurance often waited to refill medications during periodic visits to the States or bought medications online, processes that could complicate delivery. As one reported: “*We buy [medications] from Canada on the internet because they are cheaper…Canada can’t send them to Mexico, so we have to find someone in the U.S. who is planning to come down…in four to six weeks, and give them plenty of time to send it to the wrong place, not to be delivered, or to be sent back for us to spend time on the internet and the telephone trying to find them, have them sent back again, and have the person get them in time before they leave.”*

### Relationships with providers

Generous personal attention from healthcare providers was a common theme of respondents in both countries. One respondent said, *“they spend a good half hour, 45 minutes with you….It’s not like in the states where you go to see your GP and if he spends 10 minutes with you, you’re fortunate.”* Physicians were commonly reported to *“go that extra mile”* by giving out cell phone numbers and email addresses, making house calls, communicating with other providers, negotiating payment arrangements, even personally driving patients home or to the hospital. One respondent characterized his care as *“just the old, long-before-you-were-born style of medicine.”* Respondents also praised the dedication and quality of care received from home care providers.

In rare cases reports were less favorable. One interviewee reported being “*not totally thrilled with the way he [neurologist] acts towards us*,” explaining that the physician was less respectful than her doctor in the U.S. This concern prompted her to consider returning to the U.S. for healthcare. In addition, though interviewees reported widespread availability of English-speaking physicians, communication with nurses and ancillary care providers, most of whom spoke only Spanish, was often perceived as a challenge. “*Language is a problem, particularly with the nurses or attendants or receptionists*,” was a typical comment.

### Paying for healthcare

Healthcare costs were universally described as lower than in the U.S. However, virtually all interviewees expressed concern about lack of health insurance coverage in general, and the unavailability of Medicare in particular. The reported median annual out-of-pocket expenditure was $5,250 for the Mexican sample and $1,650 for the Panamanian sample. A few reported much higher out-of-pocket costs and several expressed concern about losing their savings from a high-cost illness or hospitalization. The experience of one interviewee illustrates this dilemma: *“Last January I was involved in a car accident where I was hit by a bus. The police officer at the scene told the people there that if I could afford it, to send me to (a private hospital) because my chances of survival there are going to be a lot better than my chances at the (public) hospital…I was in the hospital for two and a half months. The hospital stay cost $320,000 (U.S. dollars).”*

Reliable, comprehensive, reasonably-priced local insurance plans appeared to be largely available. Most common were limited private plans from a local hospital or medical group. One was described in this manner: *“ I don’t have a real health insurance policy … I have a very limited HMO with a local group of doctors in [nearby city] that is…very inexpensive … like maybe $200 a year… If we can find someone in that group, then we will get a discount.”* Retirees in Mexico can purchase IMSS insurance after a waiting period for approximately $300 U.S. dollars per year; however, the public system was described as confusing and many voiced concerns about quality.

Some respondents had state employee or corporate policies from the U.S. that extended benefits overseas; their reported reimbursement and satisfaction varied. One stated, for example: *“The insurance only paid about $175 of that $1,000 (hospital charge).”*

Military retirees universally complained about Tricare, the U.S. military health insurance program. Until recently, coverage for military retiree healthcare costs was available worldwide and considered excellent. However, recent reductions in payment schedules and long delays in reimbursement have caused many healthcare providers to no longer accept Tricare. One interviewee explained, “*Let’s say a doctor charges you $100 for a visit. He submits his bill to Tricare. Tricare says, we’re only going to pay him $30, and of that $30, they are only going to pay 75%.”* The result has been more expatriate retirees seeking care in the U.S. or Puerto Rico, a process that is not always practical and can lead to risky delays. One interviewee, for example, had recently attended the funeral of a friend whose Panamanian hospital stopped accepting Tricare for his dialysis, and who subsequently died while awaiting acceptance for care at a U.S. military hospital.

Retirees who qualified for Medicare were universally upset about the lack of coverage. Many felt indignation at having to continue to pay a monthly fee for Part B coverage to avoid the risk of higher premiums if they later returned to the U.S.. Every interviewee wanted to see Medicare benefits extended to retirees living abroad, many of whom believed that the U.S. would gain financially from paying for less expensive services abroad*. “It’s just costing the U.S. government more and more to have us come back, especially for basic stuff,”* one commented.

### Making healthcare choices

No respondent in the Mexican sample spoke of having chosen to locate there for healthcare reasons. In contrast, the availability, quality, and cost of healthcare were a consideration for a number of interviewees in Panama, which is booming as a medical tourism destination and has several hospitals that offer insurance plans catering to U.S. retirees (including one in Panama City that is affiliated with Johns Hopkins University). Moving abroad for healthcare reasons was particularly true for retirees in their late 50s and early 60s who had not yet qualified for Medicare, and for military veterans who were drawn by the existence of military healthcare in Panama.

Regardless of the role of healthcare in decisions to retire abroad, virtually all interviewees described the task of deciding where and how to obtain services as challenging. Contributing to this problem were not only the insurance issues described previously, but also the relative unavailability of consumer information. One respondent spoke of having comparison shopped for the best hospital to have prostate surgery in Dallas, using published statistics on complication rates and patient satisfaction, and finding no similar information available in Mexico. As a result, most retirees depended largely on informal information gathered by word-of-mouth, expatriate organizations, providers they had already met, and internet list serves and blogs.

Respondents who had Medicare reported weighing the costs of deductibles, co-pays, travel, and demands on informal caregivers when determining where and how to access services. Some maintained a house or apartment in the U.S. and returned regularly, in part to obtain medical services. Those who did not have a U.S. home base had higher travel costs. As one noted, *“you have to pay for a place to stay, food, you’ve got to rent a car, airfare, and then (hospitals) only keep you for a nano-minute…So the question is, do you go back or do you stay here and just cross your fingers?”*

When asked about long-term care, the younger and healthier retirees often reported not giving it much thought. Typical responses were “*I can’t really decide on that because I don’t know what turn my health will take*” and “*I would just have to evaluate where we are at that point.”* Those who had thought about the issue fell into two groups: those who would opt to be near family in the U.S., and those who would stay abroad and obtain in-home services. One respondent replied: *“I’d stay right where I am. I would find someone who would come in every single day or overnight depending on what I need, and I would be able to afford it.”*

## Discussion

The population of retired U.S. citizens living abroad is growing and is projected to mushroom in the coming years [2,3]. Because healthcare provision for retirees living abroad is a common concern and has undergone little systematic investigation, we interviewed retired U.S. citizens in Mexico and Panama who had significant healthcare needs. Our interviews identified many strengths regarding health services in those countries, including lower costs, availability of high quality services, personalization of care, and in-home care. Many problems were also identified, including concerns about consistent quality, reduced access to technology, decision-making challenges, inadequate insurance coverage, and a desire to see Medicare extended abroad.

As is typical of Latin America, parallel public and private healthcare systems exist in Mexico and Panama. The public systems tend to focus on the uninsured poor (though attempts to provide universal coverage are growing); resources are limited; and service quality is highly variable [19]. Persons with adequate resources, whether U.S. citizens or natives, tend to selectively utilize the private system because of its reputation for higher quality, efficiency, and highly personalized care [20], as did the vast majority of our interviewees. Our respondents’ comments were strikingly similar to those of Mexican immigrants to the U.S. who return to Mexico for health care in the private system – that doctors provide more personal care, providers appear less motivated by money, and things can get done faster [20].

Indeed, a particularly striking theme from our interviews was high satisfaction with physician providers, who were often favorably compared with those in the U.S. Interviewees frequently reported providers taking a personal interest in patients, routinely providing long appointments, and being readily available. Possible contributing factors could include true international differences in practice style or differential treatment of U.S. retirees, perhaps in part because they pay privately. Whatever the explanation, this finding is in contrast to U.S. healthcare, which, in comparison of health systems in six developed countries, was rated among the lowest in patient-centeredness, access, and efficiency [21]. Home care was another area of high satisfaction, with many respondents extolling the ready availability of high quality, affordable providers. Considering that the vast majority of U.S. seniors would prefer home-based services to institutional long-term care, the existence of inexpensive quality home care could constitute a significant attraction for retirement to Mexico or Panama [22].

Difficulty choosing where and from whom to obtain care was widely reported, with retirees needing to network extensively to make choices. In particular, a lack of available public data on provider credentials, quality, and outcomes was noted. This situation is easily remedied; one solution would be for host communities to better organize to address the informational needs of immigrant retirees [3,23].

The median annual out-of-pocket medical cost was $5,250 for the Mexican sample and $1,650 for the Panamanian sample. One explanation for the marked difference could have included differences in health status, as there was a nonsignificant trend for the Mexican sample to be older and more impaired (Table 1). Other possible contributing factors could be more insurance coverage (e.g., because more of the Panamanian sample were ex-military on Tricare) and Panama’s discount program for pensioners, which includes 15% off hospital charges and 20% off physician consultations [24].

Virtually all interviewees reported a desire to see Medicare extended abroad. Unfortunately, the vast differences between the healthcare systems, the challenges experienced by Tricare providing international coverage, and a desire by Medicare to contain expenses all mitigate against this development [25-27]. Given the barriers to Medicare extension abroad, feasible alternatives must be better developed to meet the needs of current and future emigrant retirees. Two attractive options are private insurance plans that focus on catastrophic expenses with high deductibles, and community risk pools [28,29]. Another option would be restructuring Medicare to provide cash payments and medical savings accounts; such a program would be more readily exported abroad but would provide inadequate protection against catastrophic costs. Finally, it is possible that financial pressures will lead Medicare to explore the cost-effectiveness of overseas programs for high-cost patients, using research and demonstration waivers, as a way of opening the door for Medicare payment abroad.

While it is far from clear how new health care options for U.S. retirees in Latin America will evolve, the increasing internationalization of healthcare makes it highly likely that they will emerge [13]. In addition to the current and anticipated future growth of retirement abroad, the rapid growth of medical tourism is a strong force encouraging system change [30,31]. As of 2008, 63 medical tourism companies were operating in the United States, the majority of which had been founded after 2004 [31]. In response to this trend, moves to provide better assurance of care quality are growing. For example, Joint Commission International, an extension of the main U.S. healthcare accrediting agency, currently accredits seven hospitals and health systems in Mexico and two in Panama [32].

Major limitations of the study include the purposive nature of the sample and the respondents’ limited ability to judge issues related to quality of care received. Since no census of retirees exists, the sampling method we used is typical of that employed in interview studies of migrant retirees [5,10]. Such a method limits drawing generalizations from results, however, so our conclusions should be considered hypothesis-generating rather than definitive. Whether the persons interviewed were aware of or capable of judging care quality is another potential limitation. This is especially noteworthy because at least some of the higher costs of health care in developed countries such as the U.S. and Canada are designed to prevent low-incidence, high-impact adverse events, such as medication overprescribing [21] and the spread of antibiotic-resistant infections [33].

## Conclusions

The healthcare needs of retired U.S. citizens who choose to live abroad are likely to affect increasing numbers of older Americans in the future. Retirees living abroad report a host of dilemmas regarding gaps in services, healthcare choices and insurance availability. Without attention from the policy, medical, and business communities, the problem of providing medical care to this growing population will become increasingly urgent.

## Competing interests

The authors declare that they have no competing interests.

## Authors’ contributions

PDS had full access to all of the data in the study and takes responsibility for the integrity of the data and the accuracy of the data analysis. *Study concept and design:* PDS, SZ, LWC. *Acquisition of data:* PDS, LWC, BEH. *Analysis and interpretation of data:* PDS, SZ, LWC, BEH. *Drafting of the manuscript:* PDS, SZ, LWC, BEH. *Critical revision of the manuscript for important intellectual content:* PDS, SZ, LWC, BEH. *Statistical analysis:* LWC. *Obtained funding:* PDS, BEH. *Administrative, technical, or material support:* LWC. *Study supervision:* PDS, LWC. *Final approval for publication:* PDS, SZ, LWC, BEH. All authors read and approved the final manuscript.

## Pre-publication history

The pre-publication history for this paper can be accessed here:

http://www.biomedcentral.com/1472-6963/13/411/prepub
